# Sudden Cardiac Arrest Associated with Hemodialysis: A Community-Based Study

**DOI:** 10.34067/KID.0000000705

**Published:** 2025-01-17

**Authors:** Thien Tan Tri Tai Truyen, Audrey Uy-Evanado, Lauri Holmstrom, Kyndaron Reinier, Harpriya Chugh, Jonathan Jui, Charles A. Herzog, Sumeet S. Chugh

**Affiliations:** 1 Center for Cardiac Arrest Prevention, Department of Cardiology, Smidt Heart Institute, Cedars-Sinai Health System, Los Angeles, California; 2 Department of Emergency Medicine, Oregon Health and Science University, Portland, Oregon; 3 Division of Cardiology, Department of Medicine, Hennepin Healthcare/University of Minnesota, Minneapolis, Minnesota

**Keywords:** cardiovascular, cardiovascular disease, cardiovascular events, chronic hemodialysis, CKD, chronic kidney failure, ESKD

## Abstract

**Key Points:**

- Around 25% of sudden cardiac arrest (SCA) cases among dialysis patients were associated with the dialysis procedure, a rate nearly three times higher than expected by chance.
- SCA events were more likely to occur on dialysis days, especially after long interdialytic periods, such as on Mondays and Tuesdays.
- A significant portion (23.4%) of SCA events associated with dialysis occurred within the first hour postdialysis, highlighting the need for careful monitoring.

**Background:**

Individuals with ESKD may be at increased risk of sudden cardiac arrest (SCA) associated with dialysis therapy. However, community-based studies with comprehensive adjudication of SCA are lacking.

**Methods:**

We conducted a community-based study using a case-case study design in a US population of approximately 1 million. All SCA cases with CKD were ascertained prospectively (2002–2020). We reviewed emergency medical services narratives and archived medical records from regional hospitals to capture patients' dialysis history, schedules, and the timing of SCA events in relation to dialysis sessions. Among those on regular hemodialysis, individuals who suffered SCA during hemodialysis or within an hour after completing hemodialysis (intradialytic/immediate posthemodialysis [IIHD]) were compared to cases with SCA at other times (non-IIHD). Noncompliant individuals or those intolerant of dialysis were excluded.

**Results:**

Of 1023 SCA cases with CKD, 195 (19.1%) were undergoing regular scheduled hemodialysis. Among these cases, 24.1% were IIHD SCA, while 75.9% occurred non-IIHD. The incidence of SCA during dialysis was 2.9 times higher than expected by chance. SCA events were more likely to occur on dialysis days (65.3% of events) versus 34.7% events on the four off dialysis days (*P* < 0.001). IIHD SCA had higher serum sodium (138.9±4.8 versus 135.5±5.5 mmol/L, *P* = 0.005), lower serum potassium (3.6±0.7 versus 5.6±1.6 mmol/L, *P* < 0.001), and higher bicarbonate levels (25.9±6.6 versus 20.2±5.5 mmol/L, *P* < 0.001) compared with their non-IIHD SCA counterparts. Regarding resuscitation details, IIHD SCA had a higher percentage of shockable rhythm (46.5% versus 32.4%, *P* = 0.09), witnessed collapse (85.1% versus 53.4%, *P* < 0.001), bystander cardiopulmonary resuscitation (72.3% versus 37.9%, *P* < 0.001), return of spontaneous circulation (66.0% versus 42.5%, *P* = 0.005), and survival to hospital discharge (30.4% versus 5.4%, *P* < 0.001) compared with non-IIHD SCA.

**Conclusions:**

In patients undergoing dialysis, SCA events were significantly more common on dialysis days and three-fold higher than expected by chance. We identified potential risk factors and survival outcome differences between IIHD versus non-IIHD SCA groups that warrant future investigation.

## Visual Abstract

**Figure:**
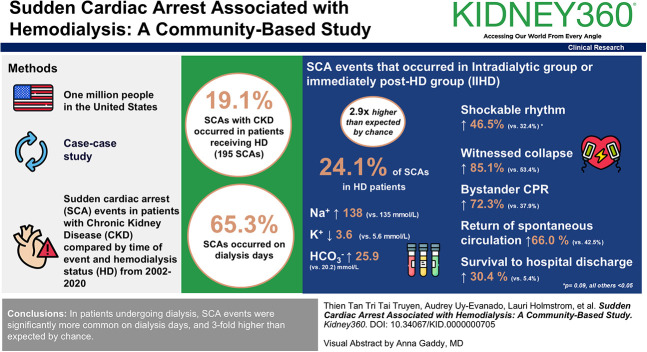

## Introduction

Sudden cardiac arrest (SCA) is one of the most lethal manifestations of human disease with <10% survival on average^1^ and remains a significant public health challenge with an estimated annual incidence of approximately 360,000 in the United States.^2–4^ Individuals suffering from CKD, particularly those with ESKD undergoing dialysis, face a significantly elevated risk of SCA.^5,6^

In the United States, approximately 37 million individuals (one in seven adults) have CKD,^7^ and roughly two in 1000 (0.2%) have ESKD and are on dialysis.^8^ Mortality among individuals on dialysis is high, and SCA accounts for a large percentage, with estimates ranging from 14%^8^ to 45%.^9–11^ The overall incidence of SCA among ESKD patients ranges from 1/100 to 10/100 person-years.^12^ Published studies estimate that SCA occurs at the rate of 4.5–7 per 100,000 hemodialysis sessions.^13–15^ Given the major public health effect and the critical need to reduce dialysis-related SCA, large community-based studies are needed that focus on the overlap between SCA and dialysis. We hypothesized that current hemodialysis techniques are associated with increased risk of SCA. Therefore, our objectives were (*1*) to estimate the proportion of SCA events associated with hemodialysis procedures among SCA individuals with ESKD undergoing hemodialysis, (*2*) to evaluate potential relationships between the timing of hemodialysis and SCA occurrences, and (*3*) to determine the potential association between hemodialysis and survival outcomes after SCA events.

## Methods

### Study Design and Population

From the Oregon Sudden Unexpected Death Study, an ongoing, prospective population-based study, we conducted a retrospective case-case study. Individuals with out-of-hospital SCA were ascertained from the Portland, Oregon, metropolitan region (catchment population of approximately 1 million) through multiple sources, namely first responders (Portland fire department and local ambulance service), the state medical examiner's office, and the emergency departments of participating local hospitals. Detailed information on circumstances, clinical history, and autopsy data (where available) were reviewed, and SCA cases of cardiac pathogenesis were identified through an in-house adjudication process conducted by three physicians. SCA was defined as a sudden, pulseless collapse due to a likely cardiac etiology, occurring rapidly after symptom onset when witnessed, or if unwitnessed, within 24 hours of the participant being last seen in their usual state of health.^4^ Cases of trauma, drowning, drug abuse, known terminal illness, or malignancy not in remission and extracardiac causes (such as pulmonary embolism) were excluded.

### Definition, Demographic Characteristics, Comorbidities, and Data on Cardiac Arrest

For this analysis, all cases of SCA with CKD from February 1, 2002, to January 31, 2020, with archived medical records from the regional medical hospitals were included. CKD was defined by a physician's diagnosis of the condition as noted in archived medical records. Subsequently, the CKD stage of each selected SCA participant was classified as the most severe stage recorded in medical records or the eGFR using the Modification of Diet in Renal Disease formula from the latest result before the onset of SCA into the following groups: stage 1 CKD (eGFR ≥90 ml/min per 1.73 m^2^), stage 2 CKD (eGFR<90 and ≥60 ml/min per 1.73 m^2^), stage 3a CKD (eGFR <60 and ≥45 ml/min per 1.73 m^2^), stage 3b CKD (eGFR <45 and ≥30 ml/min per 1.73 m^2^), stage 4 CKD (eGFR <30 and ≥15 ml/min per 1.73 m^2^), and stage 5 or ESKD (eGFR <15 ml/min per 1.73 m^2^). Among SCA individuals classified as ESKD, a study physician reviewed all available medical records of different hospitals to identify the participants receiving dialysis treatments (by looking at terms including hemodialysis and peritoneal dialysis) before their SCA event to include in our final analysis. We further reviewed all available medical records to determine the weekly schedule for hemodialysis as Tuesday—Thursday—Saturday (TTS) or Monday—Wednesday—Friday (MWF). Compliance with dialysis was also noted, and participants were determined to be either compliant or noncompliant with dialysis therapy. By reviewing the medical records, we examined the free-text narratives in the notes using keywords such as missing, intolerance, and terminate to determine noncompliance and intolerance. Noncompliance was defined as missing more than one hemodialysis session during the most recent 1-month period,^16^ and these individuals were excluded from analysis. As noted in the medical records, participants who discontinued treatment because of intolerance of the dialysis procedure were also excluded. Medical records from collaborating hospitals served as our primary information source. We did not have access to dialysis center records.

First responder prehospital reports, including the narrative of the circumstances surrounding the SCA event, were reviewed to capture the timing of the SCA event relative to hemodialysis treatments. For individuals who experienced cardiac arrest immediately after returning from dialysis, we checked their addresses, used Google Maps to identify the nearest dialysis center, and calculated the driving distance and time. They were classified as intradialytic/immediate posthemodialysis (IIHD) if the driving time was <60 minutes. Within the group receiving regularly scheduled hemodialysis, cases who suffered SCA during hemodialysis treatment or within an hour after completion (IIHD group) were compared with cases with SCA at other times (non-IIHD group). The purpose of this classification is to identify the potential causal relationship between SCA and the dialysis procedure within this population. Information on age, sex, race/ethnicity, and clinical comorbidities was obtained from hospital records. Over 90% of laboratory samples were drawn at the hospital on the patient's arrival on the day of the arrest, with the remaining samples collected within the following calendar day.

### Statistical Analysis

The data were presented using mean with SD for continuous variables and frequency with percentages for categorical variables. The *t* test and Pearson chi-square test were used to compare demographics and clinical characteristics in the IIHD versus the non-IIHD group. To evaluate whether more SCAs occurred IIHD than would be expected by chance in the hemodialysis group, we compared the observed proportion of IIHD SCA to the proportion of SCAs expected to occur IIHD on the basis of the average number of hours per week spent on dialysis in the United States reported by Tentori *et al.* as 214 minutes or 3.57 hours.^17^ For the primary analysis investigating the characteristics of IIHD and non-IIHD SCA, we included participants who had undergone prior hemodialysis, while excluding cases involving intolerance, noncompliance, and peritoneal dialysis. We conducted a multivariable analysis to evaluate potential differences between the SCA IIHD and non-IIHD SCA groups. The covariates included in the model were characteristics such as sex, age, and race, along with comorbidities relevant to cardiac arrest and those that may affect dialysis patients and procedures, including hypertension, diabetes, chronic obstructive pulmonary disease, peripheral vascular disease, anemia, coronary artery disease, and congestive heart failure. To highlight the potential relationship between the timing of dialysis and SCA occurrence, we compared the distribution of SCA cases across the weekdays between participants with CKD with and without hemodialysis selected from the same Oregon Sudden Unexpected Death Study database. For the analysis of timing, we included only cases for which we could determine their weekly dialysis schedules. A sensitivity analysis was also conducted to include individuals with intolerance or noncompliance. Significance was determined at a threshold of *P* value < 0.05, and all reported *P* values are two-sided. Statistical analyses were performed using IBM Statistical Package for Social Studies version 24.

### Ethical Considerations

This study adhered to the principles of the Declaration of Helsinki. The Institutional Review Boards of Oregon Health and Science University and all relevant hospitals/health systems have approved the study protocol, and written informed consent was obtained from all survivors of SCA before their involvement in the study.

## Results

### Study Overview and Baseline Characteristics

**Figure 1:**
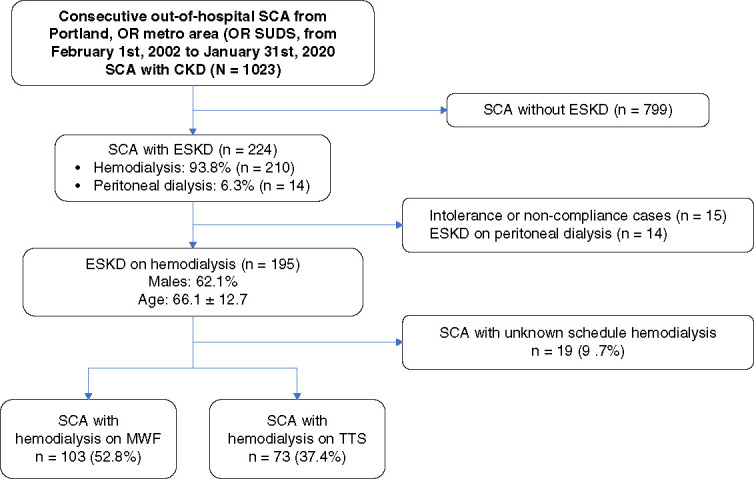
**Flow chart showing selection of SCA cases with CKD with and without dialysis.** Study participants were selected among the consecutive out-of-hospital SCA cases from Portland, Oregon metro area (ORSUDS), from February 1, 2002, to January 31, 2020. SCA individuals with ESKD known hemodialysis schedule were classified as MWF or TTS. MWF, Monday—Wednesday—Friday; ORSUDS, Oregon Sudden Unexpected Death Study; SCA, sudden cardiac arrest; TTS, Tuesday—Thursday—Saturday.

From February 1, 2002, to January 31, 2020, a total of 9765 sudden death suspected events occurred in our study region. After adjudication, 5301 cases of SCA were found. Of 1023 SCA with CKD, we found 231 (22.6%) participants with ESKD. Subsequently, individuals undergoing dialysis before SCA event included 224 participants, with 14 cases (6.3%) on peritoneal dialysis and 210 (93.8%) receiving hemodialysis (Figure 1). After excluding individuals noncompliant and intolerant with hemodialysis and those who were undergoing peritoneal dialysis, the primary analysis was restricted to individuals compliant with hemodialysis (*n*=195). The mean age (±SD) was 66.1(±12.7) years, with 62.1% being male. For the analysis of SCA distribution across weekdays, we excluded 19 individuals (9.7%) because of an inability to determine their maintenance dialysis schedules. As a result, 176 SCA participants with a confirmed weekly hemodialysis schedule were included in this analysis.

In the sensitivity analysis including noncompliant or intolerant patients, one patient was excluded because of a prior kidney transplant before the SCA event, bringing the total to 209 patients with 14 additional cases included.

### Characteristics of IIHD SCA Cases versus Non-IIHD SCA

**Figure 2:**
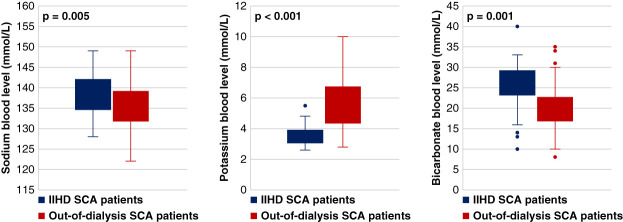
**Serum sodium, potassium, and bicarbonate levels at time of arrest in IIHD and non-IIHD SCA individuals.** IIHD, intradialytic/immediate posthemodialysis.

IIHD SCA occurred in 47 (24.1%) individuals, with 36 (18.5%) cases during the hemodialysis session and 11 (5.6%) within 1 hour of hemodialysis. Of these 11 cases, three occurred at home, while the remaining eight took place in the dialysis center, primarily in the waiting room. Compared with non-IIHD SCA, demographic and clinical characteristics (sex, age, obesity, hypertension, hyperlipidemia, chronic obstructive pulmonary disease, asthma, peripheral vascular disease, anemia, coronary artery disease, congestive heart failure, and left ventricular ejection fraction) of IIHD participants were similar except for White race, which was significantly more common in the IIHD SCA group (74.5% versus 55.8%, *P* = 0.02; Table 1). In a multivariable logistic regression model adjusted for demographics and comorbidities, White race remained significantly associated with IIHD SCA (odds ratio [OR], 2.3 [95% confidence interval (CI), 1.0 to 5.0; *P* = 0.04]). Individuals with IIHD SCA had higher serum sodium levels (138.9±4.8 versus 135.5±5.5 mmol/L, *P* = 0.005), lower potassium levels (3.6±0.7 versus 5.6±1.6 mmol/L, *P* < 0.001), and higher bicarbonate levels (25.9±6.6 versus 20.2±5.5 mmol/L, *P* < 0.001) when compared with the non-IIHD group (Figure 2). The multivariable analysis revealed similar results, indicating an OR of 1.2 (95% CI, 1.0 to 1.4; *P* = 0.01) for a one-unit increase in sodium levels and an OR of 0.2 (95% CI, 0.1 to 0.4; *P* < 0.001) for a one-unit increase in potassium levels and OR of 1.2 (95% CI, 1.1 to 1.4; *P* = 0.001) for a one-unit increase in bicarbonate levels. Similar results were found in sensitivity analysis (see Supplemental Tables 1 and 2 in Supplemental Material).

**Table 1:** Demographics and clinical characteristics of SCA cases with CKD on hemodialysis (*n*=195) from the Oregon Sudden Unexpected Death Study (2002–2020), comparing those who had their SCA during or within 1 hour of hemodialysis (IIHD) with individuals who had their SCA at other times (non-IIHD)

Characteristics	IIHD SCA (*N*=47)	Non-IIHD SCA (*N*=148)	*P* Value
Male, *n* (%)	29 (61.7)	92 (62.0)	0.96
Age, yr, mean±SD	67.5±12.2	65.6±12.8	0.37
**Race/ethnicity, *n* (%)^a^**			0.21
Asian	2 (4.3)	11 (7.5)	
Black	8 (17.0)	36 (24.5)	
Hispanic	0	10 (6.8)	
Other	2 (4.3)	8 (5.4)	
White non-Hispanic	35 (74.5)	82 (55.8)	
Missing	0	1	
**White, *n* (%)** ^a^	35 (74.5)	82 (55.8)	0.02
Missing	0	1	
**Body mass index, mean±SD** ^a^	29.0±9.5	28.4±7.9	0.69
Missing	9	22	
Diabetes, *n* (%)	29 (61.7)	115 (77.7)	0.03
Hypertension, *n* (%)	41 (87.2)	129 (87.2)	0.99
Hyperlipidemia, *n* (%)	24 (51.1)	89 (60.1)	0.27
Documented CAD, *n* (%)	29 (61.7)	88 (59.5)	0.79
Heart failure, *n* (%)	27 (57.4)	75 (50.7)	0.42
**Ejection fraction, %, mean±SD** ^a^	42.5±16.7	48.7±17.4	0.09
Missing	18	48	
COPD, *n* (%)	13 (27.7)	30 (20.3)	0.29
PVD, *n* (%)	13 (27.7)	57 (38.5)	0.18
Asthma, *n* (%)	4 (8.5)	12 (8.1)	1^b^
Anemia, *n* (%)	28 (59.6)	83 (56.1)	0.67
**Arrest blood sodium, mmol/L, mean±SD** ^a^	138.9±4.8	135.5±5.5	0.005
Missing	17	93	
**Arrest blood potassium, mmol/L, mean±SD** ^a^	3.6±0.7	5.6±1.6	<0.001
Missing	17	91	
**Arrest blood calcium, mmol/L, mean±SD** ^a^	8.6±0.9	9.2±2.2	0.07
Missing	19	97	
**Arrest blood bicarbonate, mmol/L, mean±SD** ^a^	25.9±6.6	20.2±5.5	<0.001
Missing	19	95	

CAD, coronary artery disease; COPD, chronic obstructive pulmonary disease; IIHD, intradialytic/immediate posthemodialysis; PVD, peripheral vascular disease; SCA, sudden cardiac arrest.

a For variables with missing values, proportions and *P* values were calculated with the nonmissing data used as the denominator.

b Fisher exact *t* test was used.

**Figure 3:**
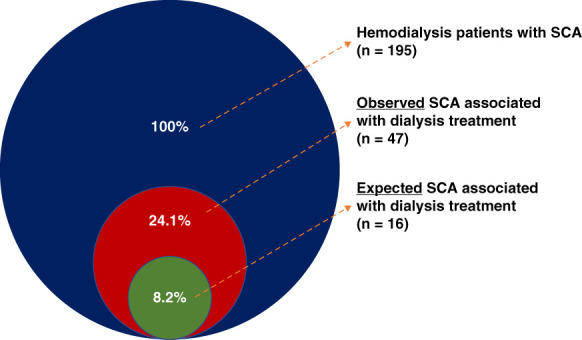
Percentage of expected and observed SCA events associated with dialysis treatment.

The average treatment time in the United States for each in-center dialysis session, conducted three times per week, is 214 minutes or 3.57 hours.^17^ We estimated that, if SCAs occurred randomly throughout the 168 hours of each week, 6.4% would occur during dialysis (3.57 hours per treatment 3/wk=10.7 hours per week) and 1.8% would occur within 1 hour postdialysis (1 hour three times per week=3 hours per week), resulting in an expected 8.2% of SCAs during the IIHD period (13.7 hours/168 total weekly hours). We observed that the proportion of SCA occurring during dialysis treatment was 2.9-fold higher than expected by chance (18.5% versus 6.4%). During the 1 hour postdialysis period, SCA prevalence was 3.1 times higher (5.6% versus 1.8%). The combined 47 cases occurring during the IIHD period (treatment and within 1 hour postdialysis) was 2.9 times higher than expected by chance (24.1 versus 8.2%; Figure 3). The percentage of IIHD SCA was 22.5% (47 out of 209), which was 2.7 times higher than expected in the sensitivity analysis.

### Timing of SCA Relative to Dialysis Schedule

**Figure 4:**
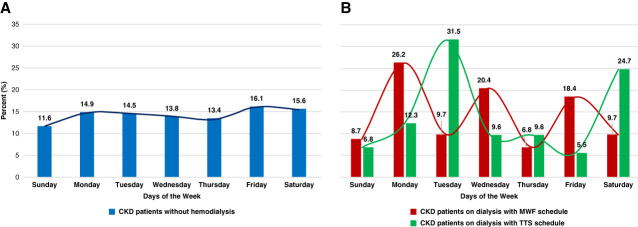
**Timing of SCA relative to dialysis schedule.** (A) Distribution of SCA events during days of the week in individuals with CKD without hemodialysis. (B) Distribution of SCA events during days of the week in individuals undergoing dialysis with MWF and TTS schedule.

Individuals on hemodialysis were more likely to have SCA events on dialysis days, including Monday (26.2%), Wednesday (20.4%), and Friday (18.4%) for MWF schedules and Tuesday (31.5%), Thursday (9.6%), and Saturday (24.7%) for TTS schedules, compared with individuals with CKD without dialysis whose percentage of SCA events occurring each day ranged from 11.7% to 16.1% (*P* < 0.001; Figure 4). The largest proportion of SCA events occurred on Mondays (26.2%) and Tuesdays (31.5%) compared with other days of the week. The sensitivity analysis provided similar results (see Supplemental Table 3).

### Differences in Resuscitation Features and Outcomes between the IIHD versus Non-IIHD Group

As expected, IIHD individuals experienced a higher incidence of cardiac arrest events in facilities, compared with their non-IIHD counterparts (93.6% versus 31.8%, *P* < 0.001). IIHD SCA cases were more likely to be witnessed during collapse (85.1% versus 53.4%, *P* < 0.001), receive bystander cardiopulmonary resuscitation (CPR; 72.3% versus 37.9%, *P* < 0.001), and present with a shockable rhythm (46.5% versus 32.4%, *P* = 0.09) than non-IIHD cases. IIHD participants had higher likelihood of return of spontaneous circulation (66.0% versus 42.5%, *P* = 0.005) and survival to hospital discharge (30.4% versus 5.4%, *P* < 0.001) when compared with non-IIHD participants (Table 2). The sensitivity analysis showed similar results (see Supplemental Table 4).

**Table 2:** SCA characteristics according to Utstein-style guidelines^37^

Characteristics	IIHD SCA (*N*=47)	Non-IIHD SCA (*N*=148)	*P* Value
**Location, *n* (%)**			<0.001
Home	3 (6.4)	92 (62.2)	
Dialysis center	44 (93.6)	4 (2.7)	
Care facilities^a^	0 (0.0)	43 (29.1)	
Public	0 (0.0)	6 (4.1)	
Other	0 (0.0)	3 (2.0)	
**Witnessed collapse, *n* (%)^b^**	40 (85.1)	78 (53.4)	<0.001
Missing	0	2	
Bystander CPR, *n* (%)	34 (72.3)	56 (37.9)	<0.001
Do not resuscitation order, *n* (%)	7 (14.9)	13 (8.8)	0.23
Automatic external defibrillator usage by bystander, *n* (%)	26 (55.3)	3 (2.0)	<0.001
**Initial presenting rhythm, *n* (%)^b^**			0.09
VFVT	20 (46.5)	44 (32.4)	
PEA/asystole	23 (53.5)	92 (67.6)	
Missing	4	12	
Resuscitation attempted, *n* (%)	40 (85.1)	135 (91.2)	0.23
**ROSC, *n* (%)** ^b^	31 (66.0)	62 (42.5)	0.005
Missing	0	2	
**Survive to hospital discharge, *n* (%)** ^b^	14 (30.4)	8 (5.4)	<0.001^c^
Missing	1	0	

CPR, cardiopulmonary resuscitation; IIHD, intradialytic/immediate post-hemodialysis; PEA, pulseless electrical activity; ROSC, return of spontaneous circulation; SCA, sudden cardiac arrest; VFVT, ventricular fibrillation and ventricular tachycardia.

a Care facilities include nursing homes and outpatient clinics.

b For variables with missing values, proportions and *P* values were calculated with the nonmissing data used as the denominator.

c Fisher exact *t* test was used.

## Discussion

In this population-based study of all SCA events occurring in a large US community (approximately 1 million residents), we report that about one-fourth of SCA cases among individuals undergoing dialysis were associated with the dialysis procedure—a proportion at least 2.9 times greater than would be expected by random chance alone. A multivariable logistic regression model showed no statistical differences in demographic characteristics and comorbidities between SCA individuals with IIHD and non-IIHD except higher prevalence of White race in the IIHD group. Further analysis of the subgroup of participants with available electrolyte levels at the time of arrest revealed that IIHD cases had higher blood levels of sodium, lower levels of potassium, and higher bicarbonate levels than their non-IIHD counterparts. Regarding the timing of SCA events relative to dialysis sessions, our findings suggest that individuals on dialysis were more prone to experiencing events on their dialysis days, particularly after extended interdialytic periods (notably Mondays and Tuesdays). Finally, regarding outcomes, IIHD individuals demonstrated better survival outcomes compared with those whose SCA occurred non-IIHD.

Our findings suggest that the nearly three-fold higher risk of SCA during dialysis therapy warrants further investigation. The lack of major differences in demographic or clinical characteristics between the two groups potentially implicates the process of dialysis as a risk determinant of SCA in patients with ESKD aligning with findings from previous studies.^14,18–21^ While specific mechanisms leading to SCA events still need to be explored, several potential determinants of lethal arrhythmia during dialysis have been discussed in the literature. These mechanisms include intravascular volume fluctuations because of predialysis hypervolemia and hypertension, accumulation of toxins, electrolyte disturbances and relatively rapid correction of these electrolyte abnormalities, variable central nervous system effects due to alternating hypotension and hypertension, and postdialytic potassium rebound.^22,23^ Although within the normal range, as expected because of the effects of dialysis, the IIHD SCA group observed in our study demonstrated lower potassium levels and higher bicarbonate levels postarrest compared with the non-IIHD SCA group. These findings align with findings from previous research^15,24–26^ and may help explain the elevated risk of SCA related to dialysis.^11^ It is conceivable that several of these interacting mechanisms could contribute to an increased risk of ventricular tachyarrhythmia and bradyarrhythmia, thereby resulting in SCA. Previous studies have found that low potassium dialysates are associated with SCA independently of predialysis serum potassium levels,^14,15^ suggesting that dialysate adjustment may influence SCA risk.

On the other hand, identification and guideline-based treatment of underlying cardiac disease are essential means to reduce the SCA burden in dialysis patients. A study by Pun *et al.* showed that using standard cardiovascular medications (*β*-blockers, calcium-channel blockers, and renin-angiotensin system inhibitors) during dialysis-related SCA was associated with improved survival rates.^27^ Although most dialysis patients have normal or only mildly reduced ejection fraction, the prevalence of structural cardiac disease with left ventricular hypertrophy and myocardial fibrosis is high^28^ and may also contribute to SCA risk.

Individuals undergoing dialysis were more likely to have an SCA event on their dialysis days. Prior studies reported a significantly higher proportion of sudden death on Mondays and Tuesdays (the first dialysis day after extended interdialytic periods) compared with other days of the week, implying that the accumulation of electrolytes, metabolites, and fluids between dialysis sessions increased the risk of lethal arrhythmias because of the weekend respite from dialysis.^14,19,21,24,29,30^ Our study not only confirmed previous findings, with SCA occurrences peaking at 26.2% on Mondays for the MWF schedule and 31.5% on Tuesdays for the TTS schedule, but also revealed a distinct pattern: dialysis days (MWF and TTS) were associated with higher rates of SCA events. For non-hemodialysis CKD participants, SCA occurrence was evenly distributed throughout the week, further supporting the hypothesis that stress induced by the dialysis procedure may seem as a risk factor for sudden cardiac death occurrence in dialysis individuals. Interestingly, another study by Krishnasamy *et al.* demonstrated that the daily variation in the cardiac death pattern was present only in in-center hemodialysis patients who received ≤3 dialysis sessions per week, while patients in peritoneal dialysis, home hemodialysis, or in-center hemodialysis >3 sessions per week did not have a similar pattern in cardiac death.^21^ Hence, considering alternative dialysis methods in high-risk individuals may reduce SCA risk.

Finally, regarding the SCA outcomes, individuals with SCA IIHD had a higher chance of rapid resuscitation. Bystander CPR rates were higher compared with their non-IIHD counterparts because of location of events in care facilities. Accordingly, there was a higher probability of initial shockable rhythm and better survival outcomes. While risk of SCA during dialysis is greater, survival from SCA after resuscitation is also higher. Our results align with previous evidence indicating that cardiac arrests in outpatient dialysis centers (particularly events happening during or after dialysis) are more likely to involve shockable rhythms (62%–67%) and result in better outcomes (38% overall survival to hospital discharge).^20,31,32^ Our results revealed another aspect of the complex picture of SCA occurring during dialysis, specifically caused by ventricular arrhythmia. Although previous studies have reported a very low incidence of ventricular arrhythmia in patients undergoing hemodialysis,^30,33–35^ our findings suggest that when it does occur, there might be a higher likelihood of it leading to SCA. These findings support deployment of automated external defibrillators in all dialysis units and emphasize the need to address their possible current underutilization.^36^ Finally, it is worth noting that the low rate of bystander CPR in our study aligns with previous findings from Pun *et al.* study using the cardiac arrest registry to enhance survival data registry.^36^ Our findings provide a possible underlying reason: the high percentage of SCA events occurring within 1 hour postdialysis (11 of 47 cases, or 23.4%), a critical period during which these events may go unnoticed by health care staff at dialysis centers. These results underscore the importance of careful monitoring, particularly within the first hour after a dialysis session.

There are some limitations of this study that should be considered when interpreting these findings. First, it is important to note that our research is based on a large community-based observational analysis. Although we used multivariable regression models to reduce confounding bias, some residual confounders may still exist. Second, the diagnosis and classification of CKD, ESKD, and maintenance hemodialysis were made by reviewing all available medical records from participating hospitals. Dialysis clinic notes were not available because of our primary study design, which disallowed examination of important known dialytic risk factors, including other electrolytes, ultrafiltration rate, and dialysis time. This also led to incomplete data about dialysis schedules, which could have biased results of the day of week analysis. The intolerance and noncompliance of SCA participants were defined on the basis of the narrative and medical records, which may not reflect accurately the status of those patients. The size of the prevalent population of hemodialysis patients within the study catchment area was not available. Third, all clinical and laboratory data may not be uniformly available for all individuals, noting that sudden cardiac deaths in dialysis centers were more likely to have electrolyte measurements compared to those occurring outside of dialysis centers. In addition, there are no similarly timed posthemodialysis laboratory values available for the out-of-clinic cohort, limiting the ability to directly compare laboratory values between these groups. Finally, owing to the nature of out-of-hospital SCA, the initial rhythm data may be influenced by delays in emergency medical services (EMS) arrival, rather than reflecting the definitive underlying rhythm at the time of the cardiac arrest. Despite these limitations, our study has strengths that are worth noting. First, our study design, which includes strict and systematic adjudication of SCA, enables a more accurate evaluation of the burden of SCA in dialysis patients in community compared with previous studies that relied on implantable cardioverter-defibrillator codes to define SCA. Second, our study included detailed EMS records, which allowed for the accurate assessment of resuscitation information. Finally, by combining these records with comprehensive medical data from regional hospitals, we were able to enhance the robustness of the EMS records in evaluating the comorbidities of individuals who experienced SCA.

In this population-based study, there was a significant association between dialysis sessions and a higher occurrence of SCA events. SCA events IIHD seem to be significantly different compared with SCA Non-IIHD both for mechanisms as well as survival outcomes. The hemodialysis procedure is a potential SCA risk factor in the dialysis population. Therefore, to improve mortality outcomes for individuals undergoing dialysis, comprehensive investigations of the association between hemodialysis and SCA are warranted.

## Supplementary Material

SUPPLEMENTARY MATERIAL

## Data Availability

All data are included in the manuscript and/or supporting information. Partial restrictions to the data and/or materials apply. The data in this manuscript are from an ongoing study, and there is currently no IRB-approved mechanism by which this data will be deposited in a public repository. All analytical methods are included in this published article. Deidentified participant data will be made available after publication upon reasonable request to the corresponding author, following approval of a proposal and a signed data use agreement.

## Supplemental Material

Sensitivity analysis of population including the intolerance or non-compliance cases

Supplemental Table 1. Demographics and clinical characteristics of SCA cases.

Supplemental Table 2. Laboratory at time of arrest of the patients who had their SCA during or within 1 hour of hemodialysis (IIHD) to individuals who had their SCA at other times (out-of-dialysis).

Supplemental Table 3. Distribution patterns of sudden cardiac death in dialysis patients on MWF and TTS schedules.

Supplemental Table 4. SCA characteristics of the patients who had their SCA during or within 1 hour of hemodialysis (IIHD) to individuals who had their SCA at other times (out-of-dialysis) according to Utstein-style guidelines.

## References

[1] Nichol G Thomas E Callaway CW, . Regional variation in out-of-hospital cardiac arrest incidence and outcome. JAMA. 2008;300(12):1423–1431. doi:10.1001/jama.300.12.1423 18812533 PMC3187919

[2] Adabag AS Luepker RV Roger VL Gersh BJ. Sudden cardiac death: epidemiology and risk factors. Nat Rev Cardiol. 2010;7(4):216–225. doi:10.1038/nrcardio.2010.3 20142817 PMC5014372

[3] Chugh SS Reinier K Teodorescu C, . Epidemiology of sudden cardiac death: clinical and research implications. Prog Cardiovasc Dis. 2008;51(3):213–228. doi:10.1016/j.pcad.2008.06.003 19026856 PMC2621010

[4] Fishman GI Chugh SS Dimarco JP, . Sudden cardiac death prediction and prevention: report from a National Heart, Lung, and Blood Institute and Heart Rhythm Society Workshop. Circulation. 2010;122(22):2335–2348. doi:10.1161/CIRCULATIONAHA.110.976092 21147730 PMC3016224

[5] Pun PH Smarz TR Honeycutt EF Shaw LK Al-Khatib SM Middleton JP. Chronic kidney disease is associated with increased risk of sudden cardiac death among patients with coronary artery disease. Kidney Int. 2009;76(6):652–658. doi:10.1038/ki.2009.219 19536082 PMC2990680

[6] Turakhia MP Blankestijn PJ Carrero JJ, . Chronic kidney disease and arrhythmias: conclusions from a Kidney Disease: Improving Global Outcomes (KDIGO) Controversies Conference. Eur Heart J. 2018;39(24):2314–2325. doi:10.1093/eurheartj/ehy060 29522134 PMC6012907

[7] National Institute of Diabetes and Digestive and Kidney Diseases. Kidney Disease Statistics for the United States, 2022. Accessed August 30, 2022. https://www.niddk.nih.gov/health-information/health-statistics/kidney-disease

[8] United States Renal Data System. 2020 USRDS Annual Data Report: Epidemiology of Kidney Disease in the United States. National Institutes of Health, US Department of Health and Human Services, 2020.

[9] de Bie MK van Dam B Gaasbeek A, . The current status of interventions aiming at reducing sudden cardiac death in dialysis patients. Eur Heart J. 2009;30(13):1559–1564. doi:10.1093/eurheartj/ehp185 19465437

[10] Herzog CA. Cardiac arrest in dialysis patients: approaches to alter an abysmal outcome. Kidney Int. 2003;63(84):S197–S200. doi:10.1046/j.1523-1755.63.s84.17.x 12694343

[11] Makar MS Pun PH. Sudden cardiac death among hemodialysis patients. Am J Kidney Dis. 2017;69(5):684–695. doi:10.1053/j.ajkd.2016.12.006 28223004 PMC5457912

[12] Ramesh S Zalucky A Hemmelgarn BR, . Incidence of sudden cardiac death in adults with end-stage renal disease: a systematic review and meta-analysis. BMC Nephrol. 2016;17(1):78. doi:10.1186/s12882-016-0293-8 27401469 PMC4940956

[13] Shamseddin MK Parfrey PS. Sudden cardiac death in chronic kidney disease: epidemiology and prevention. Nat Rev Nephrol. 2011;7(3):145–154. doi:10.1038/nrneph.2010.191 21283136

[14] Karnik JA Young BS Lew NL, . Cardiac arrest and sudden death in dialysis units. Kidney Int. 2001;60(1):350–357. doi:10.1046/j.1523-1755.2001.00806.x 11422771

[15] Pun PH Lehrich RW Honeycutt EF Herzog CA Middleton JP. Modifiable risk factors associated with sudden cardiac arrest within hemodialysis clinics. Kidney Int. 2011;79(2):218–227. doi:10.1038/ki.2010.315 20811332

[16] Ozen N Cinar FI Askin D Mut D Turker T. Nonadherence in hemodialysis patients and related factors: a multicenter study. J Nurs Res. 2019;27(4):e36. doi:10.1097/jnr.0000000000000309 30720548 PMC6641098

[17] Tentori F Zhang J Li Y, . Longer dialysis session length is associated with better intermediate outcomes and survival among patients on in-center three times per week hemodialysis: results from the Dialysis Outcomes and Practice Patterns Study (DOPPS). Nephrol Dial Transplant. 2012;27(11):4180–4188. doi:10.1093/ndt/gfs021 22431708 PMC3529546

[18] Bleyer AJ Russell GB Satko SG. Sudden and cardiac death rates in hemodialysis patients. Kidney Int. 1999;55(4):1553–1559. doi:10.1046/j.1523-1755.1999.00391.x 10201022

[19] Bleyer AJ Hartman J Brannon PC Reeves-Daniel A Satko SG Russell G. Characteristics of sudden death in hemodialysis patients. Kidney Int. 2006;69(12):2268–2273. doi:10.1038/sj.ki.5000446 16672908

[20] Davis TR Young BA Eisenberg MS Rea TD Copass MK Cobb LA. Outcome of cardiac arrests attended by emergency medical services staff at community outpatient dialysis centers. Kidney Int. 2008;73(8):933–939. doi:10.1038/sj.ki.5002749 18172435

[21] Krishnasamy R Badve SV Hawley CM, . Daily variation in death in patients treated by long-term dialysis: comparison of in-center hemodialysis to peritoneal and home hemodialysis. Am J Kidney Dis. 2013;61(1):96–103. doi:10.1053/j.ajkd.2012.07.008 22901771

[22] Rhee CM Ayus JC Kalantar-Zadeh K. Hyponatremia in the dialysis population. Kidney Int Rep. 2019;4(6):769–780. doi:10.1016/j.ekir.2019.02.012 31194059 PMC6551474

[23] Bansal S Pergola PE. Current management of hyperkalemia in patients on dialysis. Kidney Int Rep. 2020;5(6):779–789. doi:10.1016/j.ekir.2020.02.1028 32518860 PMC7270720

[24] Genovesi S Valsecchi MG Rossi E, . Sudden death and associated factors in a historical cohort of chronic haemodialysis patients. Nephrol Dial Transplant. 2009;24(8):2529–2536. doi:10.1093/ndt/gfp104 19293137

[25] Jadoul M Thumma J Fuller DS, . Modifiable practices associated with sudden death among hemodialysis patients in the Dialysis Outcomes and Practice Patterns Study. Clin J Am Soc Nephrol. 2012;7(5):765–774. doi:10.2215/CJN.08850811 22403271 PMC3338277

[26] Krahn RE Tulowitzki R Gudleski GD, . Effect of bicarbonate-buffered dialysate on ventricular arrhythmias in hemodialysis patients. Am J Nephrol. 2019;49(1):74–80. doi:10.1159/000495846 30602157

[27] Pun PH Lehrich RW Smith SR Middleton JP. Predictors of survival after cardiac arrest in outpatient hemodialysis clinics. Clin J Am Soc Nephrol. 2007;2(3):491–500. doi:10.2215/CJN.02360706 17699456

[28] Jankowski J Floege J Fliser D Böhm M Marx N. Cardiovascular disease in chronic kidney disease: pathophysiological insights and therapeutic options. Circulation. 2021;143(11):1157–1172. doi:10.1161/circulationaha.120.050686 33720773 PMC7969169

[29] Foley RN Gilbertson DT Murray T Collins AJ. Long interdialytic interval and mortality among patients receiving hemodialysis. New Engl J Med. 2011;365(12):1099–1107. doi:10.1056/NEJMoa1103313 21992122

[30] Wong MC Kalman JM Pedagogos E, . Temporal distribution of arrhythmic events in chronic kidney disease: highest incidence in the long interdialytic period. Heart Rhythm. 2015;12(10):2047–2055. doi:10.1016/j.hrthm.2015.06.033 26111801

[31] Becker L Eisenberg M Fahrenbruch C Cobb L. Cardiac arrest in medical and dental practices: implications for automated external defibrillators. Arch Intern Med. 2001;161(12):1509–1512. doi:10.1001/archinte.161.12.1509 11427098

[32] Herzog CA. Cardiac arrest in dialysis patients: taking a small step. Semin Dial. 2004;17(3):184–185. doi:10.1111/j.0894-0959.2004.17318.x 15144539

[33] Roy-Chaudhury P Tumlin JA Koplan BA, . Primary outcomes of the Monitoring in Dialysis Study indicate that clinically significant arrhythmias are common in hemodialysis patients and related to dialytic cycle. Kidney Int. 2018;93(4):941–951. doi:10.1016/j.kint.2017.11.019 29395340

[34] Roberts PR Zachariah D Morgan JM, . Monitoring of arrhythmia and sudden death in a hemodialysis population: the CRASH-ILR Study. PLoS One. 2017;12(12):e0188713. doi:10.1371/journal.pone.0188713 29240772 PMC5730159

[35] Sacher F Jesel L Borni-Duval C, . Cardiac rhythm disturbances in hemodialysis patients: early detection using an implantable loop recorder and correlation with biological and dialysis parameters. JACC Clin Electrophysiol. 2018;4(3):397–408. doi:10.1016/j.jacep.2017.08.002 30089568

[36] Pun PH Dupre ME Starks MA, . Outcomes for hemodialysis patients given cardiopulmonary resuscitation for cardiac arrest at outpatient dialysis clinics. J Am Soc Nephrol. 2019;30(3):461–470. doi:10.1681/ASN.2018090911 30733235 PMC6405155

[37] Bray JE Grasner JT Nolan JP, . Cardiac arrest and cardiopulmonary resuscitation outcome reports: 2024 update of the Utstein out-of-hospital cardiac arrest registry template. Circulation. 2024;150(9):e203–e223. doi:10.1161/cir.0000000000001243 39045706

