# Supplementary material for: Sudden Cardiac Arrest Associated with Hemodialysis: A Community-Based Study
Source: Kidney360. 2025 Jan 17;6(5):805–13. doi: 10.34067/KID.0000000705 (PMC12136628; doi:10.34067/KID.0000000705)
Supplement: SUPPLEMENTARY MATERIAL [file kidney360-6-805-s001.pdf]

ASN Journal Disclosure Form

As per ASN journal policy, I have disclosed any financial relationships or commitments I have held in the past 36 months as included below. I have listed my Current Employer below to indicate there is a relationship requiring disclosure. If no relationship exists, my Current Employer is not listed.

H. Chugh has nothing to disclose.

I understand that the information above will be published within the journal article, if accepted, and that failure to comply and/or to accurately and completely report the potential financial conflicts of interest could lead to the following: 1) Prior to publication, article rejection, or 2) Post-publication, sanctions ranging from, but not limited to, issuing a correction, reporting the inaccurate information to the authors' institution, banning authors from submitting work to ASN journals for varying lengths of time, and/or retraction of the published work.

Name: Harpriya Chugh
Manuscript ID: K360-2024-000748R3
Manuscript Title: Sudden Cardiac Arrest Associated with Hemodialysis – A community-based study
Date of Completion: January 9, 2025
Disclosure Updated Date: January 9, 2025

ASN
American Society of Nephrology
JASN
Journal of the American Society of Nephrology
KIDNEY360
Accessing Our World From Every Angle
CJASN
Clinical Journal of the American Society of Nephrology

ASN Journal Disclosure Form

As per ASN journal policy, I have disclosed any financial relationships or commitments I have held in the past 36 months as included below. I have listed my Current Employer below to indicate there is a relationship requiring disclosure. If no relationship exists, my Current Employer is not listed.

S. Chugh has nothing to disclose.

I understand that the information above will be published within the journal article, if accepted, and that failure to comply and/or to accurately and completely report the potential financial conflicts of interest could lead to the following: 1) Prior to publication, article rejection, or 2) Post-publication, sanctions ranging from, but not limited to, issuing a correction, reporting the inaccurate information to the authors' institution, banning authors from submitting work to ASN journals for varying lengths of time, and/or retraction of the published work.

Name: Sumeet S Chugh
Manuscript ID: K360-2024-000748R3
Manuscript Title: Sudden Cardiac Arrest Associated with Hemodialysis – A community-based study
Date of Completion: December 17, 2024
Disclosure Updated Date: December 17, 2024

ASN
American Society of Nephrology
JASN
Journal of the American Society of Nephrology
KIDNEY360
Accessing Our World From Every Angle
CJASN
Clinical Journal of the American Society of Nephrology

ASN Journal Disclosure Form

As per ASN journal policy, I have disclosed any financial relationships or commitments I have held in the past 36 months as included below. I have listed my Current Employer below to indicate there is a relationship requiring disclosure. If no relationship exists, my Current Employer is not listed.

A. Uy-Evanado has nothing to disclose.

I understand that the information above will be published within the journal article, if accepted, and that failure to comply and/or to accurately and completely report the potential financial conflicts of interest could lead to the following: 1) Prior to publication, article rejection, or 2) Post-publication, sanctions ranging from, but not limited to, issuing a correction, reporting the inaccurate information to the authors' institution, banning authors from submitting work to ASN journals for varying lengths of time, and/or retraction of the published work.

Name: Audrey Uy-Evanado
Manuscript ID: K360-2024-000748R3
Manuscript Title: Sudden Cardiac Arrest Associated with Hemodialysis – A community-based study
Date of Completion: December 11, 2024
Disclosure Updated Date: December 11, 2024

ASN American Society of Nephrology | JASN Journal of the American Society of Nephrology | KIDNEY360 Accessing Our World From Every Angle | CJASN Clinical Journal of the American Society of Nephrology

ASN Journal Disclosure Form

As per ASN journal policy, I have disclosed any financial relationships or commitments I have held in the past 36 months as included below. I have listed my Current Employer below to indicate there is a relationship requiring disclosure. If no relationship exists, my Current Employer is not listed.

C. Herzog reports the following:
Employer: Hennepin Healthcare System, Inc. (Non profit entity); Consultancy: AstraZeneca, Bayer, DiaMedica, Merck; Ownership Interest: Stock only (no other ownership role): Boston Scientific, General Electric, Johnson & Johnson, Merck., Pfizer; Research Funding: University of British Columbia, NHLBI/NIDDK(NIH) (Non-profit funding organizations); and Patents or Royalties: Up-To-Date (Author Royalties).

I understand that the information above will be published within the journal article, if accepted, and that failure to comply and/or to accurately and completely report the potential financial conflicts of interest could lead to the following: 1) Prior to publication, article rejection, or 2) Post-publication, sanctions ranging from, but not limited to, issuing a correction, reporting the inaccurate information to the authors' institution, banning authors from submitting work to ASN journals for varying lengths of time, and/or retraction of the published work.

Name: Charles A. Herzog
Manuscript ID: K360-2024-000748R2
Manuscript Title: "Sudden Cardiac Arrest Associated with Hemodialysis – A community-based study."
Date of Completion: December 6, 2024
Disclosure Updated Date: May 8, 2024

ASN American Society of Nephrology | JASN Journal of the American Society of Nephrology | KIDNEY360 Accessing Our World From Every Angle | CJASN Clinical Journal of the American Society of Nephrology

ASN Journal Disclosure Form

As per ASN journal policy, I have disclosed any financial relationships or commitments I have held in the past 36 months as included below. I have listed my Current Employer below to indicate there is a relationship requiring disclosure. If no relationship exists, my Current Employer is not listed.

L. Holmström has nothing to disclose.

I understand that the information above will be published within the journal article, if accepted, and that failure to comply and/or to accurately and completely report the potential financial conflicts of interest could lead to the following: 1) Prior to publication, article rejection, or 2) Post-publication, sanctions ranging from, but not limited to, issuing a correction, reporting the inaccurate information to the authors' institution, banning authors from submitting work to ASN journals for varying lengths of time, and/or retraction of the published work.

Name: Lauri Holmström
Manuscript ID: K360-2024-000748R3
Manuscript Title: Sudden Cardiac Arrest Associated with Hemodialysis – A community-based study
Date of Completion: December 11, 2024
Disclosure Updated Date: December 11, 2024

ASN JASN Journal of the American Society of Nephrology KIDNEY360 Accessing Our World From Every Angle CJASN Clinical Journal of the American Society of Nephrology

ASN Journal Disclosure Form

As per ASN journal policy, I have disclosed any financial relationships or commitments I have held in the past 36 months as included below. I have listed my Current Employer below to indicate there is a relationship requiring disclosure. If no relationship exists, my Current Employer is not listed.

J. Jui reports the following:
Employer: Oregon Health and Sciences University

I understand that the information above will be published within the journal article, if accepted, and that failure to comply and/or to accurately and completely report the potential financial conflicts of interest could lead to the following: 1) Prior to publication, article rejection, or 2) Post-publication, sanctions ranging from, but not limited to, issuing a correction, reporting the inaccurate information to the authors' institution, banning authors from submitting work to ASN journals for varying lengths of time, and/or retraction of the published work.

Name: Jonathan Jui
Manuscript ID: K360-2024-000748R2
Manuscript Title: Sudden Cardiac Arrest Associated with Hemodialysis – A community-based study
Date of Completion: December 5, 2024
Disclosure Updated Date: November 9, 2024

ASN Journal Disclosure Form

As per ASN journal policy, I have disclosed any financial relationships or commitments I have held in the past 36 months as included below. I have listed my Current Employer below to indicate there is a relationship requiring disclosure. If no relationship exists, my Current Employer is not listed.

K. Reinier reports the following:
Employer: Cedars-Sinai Health System

I understand that the information above will be published within the journal article, if accepted, and that failure to comply and/or to accurately and completely report the potential financial conflicts of interest could lead to the following: 1) Prior to publication, article rejection, or 2) Post-publication, sanctions ranging from, but not limited to, issuing a correction, reporting the inaccurate information to the authors' institution, banning authors from submitting work to ASN journals for varying lengths of time, and/or retraction of the published work.

Name: Kyndaron Reinier
Manuscript ID: K360-2024-000748R3
Manuscript Title: Sudden Cardiac Arrest Associated with Hemodialysis – A community-based study
Date of Completion: December 11, 2024
Disclosure Updated Date: December 11, 2024

ASN
American Society of Nephrology
JASN
Journal of the American Society of Nephrology
KIDNEY360
Accessing Our World From Every Angle
CJASN
Clinical Journal of the American Society of Nephrology

ASN Journal Disclosure Form

As per ASN journal policy, I have disclosed any financial relationships or commitments I have held in the past 36 months as included below. I have listed my Current Employer below to indicate there is a relationship requiring disclosure. If no relationship exists, my Current Employer is not listed.

T. Truyen reports the following:
Employer: Cedars-Sinai Medical Center

I understand that the information above will be published within the journal article, if accepted, and that failure to comply and/or to accurately and completely report the potential financial conflicts of interest could lead to the following: 1) Prior to publication, article rejection, or 2) Post-publication, sanctions ranging from, but not limited to, issuing a correction, reporting the inaccurate information to the authors' institution, banning authors from submitting work to ASN journals for varying lengths of time, and/or retraction of the published work.

Name: Thien Tan Tri Tai Truyen
Manuscript ID: K360-2024-000748R2
Manuscript Title: Sudden Cardiac Arrest Associated with Hemodialysis – A community-based study
Date of Completion: December 5, 2024
Disclosure Updated Date: December 5, 2024
