# Supplementary material for: Sudden Cardiac Arrest Associated with Hemodialysis: A Community-Based Study
Source: Kidney360. 2025 Jan 17;6(5):805–13. doi: 10.34067/KID.0000000705 (PMC12136628; doi:10.34067/KID.0000000705)
Supplement: SUPPLEMENTARY MATERIAL [file kidney360-6-805-s002.pdf]

Supplemental Appendix

Table of Contents

Sensitivity analysis of population including the intolerance or non-compliance cases 3

Supplemental Table 1. Demographics and clinical characteristics of SCA cases with CKD on hemodialysis (n=209) from the Oregon SUDS study (2002-2020), comparing those who had their SCA during or within 1 hour of hemodialysis (IIHD) to individuals who had their SCA at other times (Out-of-dialysis) 5

Supplemental Table 2. Laboratory at time of arrest of the patients who had their SCA during or within 1 hour of hemodialysis (IIHD) to individuals who had their SCA at other times (Out-of-dialysis) 7

Supplemental Table 3. Distribution Patterns of Sudden Cardiac Death in Dialysis Patients on Monday-Wednesday-Friday and Tuesday-Thursday-Saturday Schedules 8

Supplemental Table 4. Sudden cardiac arrest characteristics of the patients who had their SCA during or within 1 hour of hemodialysis (IIHD) to individuals who had their SCA at other times (Out-of-dialysis) according to Utstein-style guidelines 9

Supplemental Table 5. Baseline characteristics of non-dialysis CKD groups 11

Sensitivity analysis of population including the intolerance or non-compliance cases

For this sensitivity analysis, we included patients who were excluded from the primary analysis due to intolerance or non-compliance. However, one patient was ruled out from this analysis due to a kidney transplant prior to SCA event, resulting in an additional 14 cases included in the analysis.

In total, we have 209 patients with mean age of 65.6 ± 12.9 and 129 (61.7%) males. The percentage of IIHD SCA was 22.5% (47/209) which was 2.7 times higher than expected (8.2%). General characteristics of this population were detailed in Supplemental Table 1 and Supplemental Table 2.

By including the 14 cases of intolerance and non-compliance, the analysis consisted of 107 cases on the MWF schedule and 83 cases on the TTS schedule. The number of arrests per weekday in these two groups is shown in Supplemental Table 3. A Chi-Square Goodness-of-Fit Test was conducted to determine whether the distribution of cases per weekday significantly differed from a random, equal distribution. Consistent with the primary analysis, individuals on hemodialysis were found to be more likely to experience SCA events on dialysis days, with $p < 0.001$.

Resuscitation characteristics were included in Supplemental Table 4 which showed similar results as primary analysis that IIHD experienced a higher incidence of cardiac arrest events in care facilities compared to those who experienced events outside of dialysis. IIHD SCA cases were more likely to be witnessed at the time of collapse,

receive bystander CPR, and present with a shockable rhythm than Out-of-dialysis cases. Additionally, IIHD subjects showed a higher likelihood of achieving return of spontaneous circulation and survival to hospital discharge compared to their Out-of-dialysis counterparts.

Supplemental Table 1. Demographics and clinical characteristics of SCA cases with CKD on hemodialysis (n=209) from the Oregon SUDS study (2002-2020), comparing those who had their SCA during or within 1 hour of hemodialysis (IIHD) to individuals who had their SCA at other times (Out-of-dialysis)

	IIHD SCA<br>N = 47	Out-of-dialysis SCA<br>N = 162	p-value
Male, n (%)	29 (61.7)	100 (61.7)	1.00
Age, (mean ± SD), year	67.5 ± 12.2	65.0 ± 13.1	0.24
Race/ethnicity, n (%) *			0.14
White Non-Hispanic	35 (74.5)	90 (55.9)	
Black	8 (17.0)	40 (24.8)	
Asian	2 (4.3)	11 (6.8)	
Hispanic	0	12 (7.5)	
Other	2 (4.3)	8 (5.0)	
Missing		1	
White, n (%) *	35 (74.5)	90 (55.6)	0.02
Missing		1	
Body mass index (mean ± SD) *	29.0 ± 9.5	28.4 ± 7.9	0.69
Missing	9	22	
Diabetes, n (%)	29 (61.7)	123 (75.9)	0.05

Hypertension, n (%)	41 (87.2)	139 (85.8)	0.80
Hyperlipidemia, n (%)	24 (51.1)	94 (58.0)	0.39
Documented CAD, n (%)	29 (61.7)	94 (58.0)	0.65
Heart failure, n (%)	27 (57.4)	84 (51.9)	0.50
Ejection fraction (mean ± SD) *	42.5 ± 16.7	48.2 ± 17.2	0.11
Missing	18	52	
COPD, n (%)	13 (27.7)	33 (20.4)	0.29
PVD, n (%)	13 (27.7)	63 (38.9)	0.16
Asthma, n (%)	4 (8.5%)	17 (10.5)	0.79†
Anemia, n (%)	28 (59.6)	92 (56.8)	0.73

*For variables with missing values, proportions and p values were calculated with the non-missing data used as the denominator.

†Fisher exact t-test was used.

SCA: Sudden cardiac arrest; SD: standard deviation; CAD: Coronary artery disease; COPD: Chronic obstructive pulmonary disease; PVD: Peripheral vascular disease

Supplemental Table 2. Laboratory at time of arrest of the patients who had their SCA during or within 1 hour of hemodialysis (IIHD) to individuals who had their SCA at other times (Out-of-dialysis)

	IIHD SCA<br>N = 47	Out-of-dialysis SCA<br>N = 162	p – value	Adjusted odds ratio (95% CI) *
Arrest blood sodium, mean ± SD, mmol/L †<br>Missing	138.9 ± 4.8<br>17	135.8 ± 5.6<br>104	0.009	1.2 (1.0 – 1.3)
Arrest blood potassium, mean ± SD, mmol/L †<br>Missing	3.6 ± 0.7<br>17	5.6 ± 1.6<br>104	<0.001	0.2 (0.1 – 0.4)
Arrest blood calcium, mean ± SD, mmol/L †<br>Missing	8.6 ± 0.9<br>19	9.2 ± 2.2<br>108	0.06	0.7 (0.4 – 1.1)
Arrest blood bicarbonate, mean ± SD, mmol/L †<br>Missing	25.9 ± 6.6<br>19	20.3 ± 5.4<br>106	<0.001	1.2 (1.1 – 1.4)

* Odds ratio adjusted by multivariable analysis including sex, age, and race, along with comorbidities including hypertension, diabetes, chronic obstructive pulmonary disease, peripheral vascular disease, anemia, coronary artery disease, and congestive heart failure.

†For variables with missing values, proportions and p values were calculated with the non-missing data used as the denominator.

SD: Standard deviation.

Supplemental Table 3. Distribution Patterns of Sudden Cardiac Death in Dialysis Patients on Monday-Wednesday-Friday and Tuesday-Thursday-Saturday Schedules

Day of arrest, n (%)	MWF schedule	TTS Schedule
Monday	28 (26.2)	12 (14.5)
Tuesday	11 (10.3)	25 (30.1)
Wednesday	21 (19.6)	8 (9.6)
Thursday	9 (8.4)	7 (8.4)
Friday	19 (17.8)	5 (6.0)
Saturday	10 (9.3)	20 (24.1)
Sunday	9 (8.4)	6 (7.2)

Supplemental Table 4. Sudden cardiac arrest characteristics of the patients who had their SCA during or within 1 hour of hemodialysis (IIHD) to individuals who had their SCA at other times (Out-of-dialysis) according to Utstein-style guidelines

	During-dialysis SCA N = 47	Out-of-dialysis SCA N = 162	p-value
Location, n (%)			<0.001
Home	3 (6.4)	99 (61.1)	
Care facilities†	44 (93.6)	53 (32.7)	
Public	0 (0.0)	7 (4.3)	
Other	0 (0.0)	3 (1.9)	
Witnessed collapse, n (%) *	40 (85.1)	85 (53.1)	<0.001
Missing	0	2	
Bystander CPR, n (%)	34 (72.3)	63 (38.9)	<0.001
Initial presenting rhythm, n (%) *			0.10
VFVT	20 (46.5)	49 (32.7)	

PEA/Asystole<br>Missing	23 (53.5)<br>4	101 (67.3)<br>12	
ROSC, n (%) *<br>Missing	31 (66.0)<br>0	67 (41.9)<br>2	0.004
STHD, n (%) *<br>Missing	14 (30.4)<br>1	9 (5.6)<br>0	<0.001

* For variables with missing values, proportions and p values were calculated with the non-missing data used as the denominator.

† Care facilities includes nursing homes, dialysis centers, and outpatient clinics.

SCA: Sudden cardiac arrest; CPR: Cardiopulmonary resuscitation, VFVT: Ventricular fibrillation and ventricular tachycardia; PEA: Pulseless electrical activity; ROSC: Return of spontaneous circulation; STHD: Survive to hospital discharge.

Supplemental Table 5. Baseline characteristics of non-dialysis CKD groups

	Non-dialysis CKD group<br>N = 799
Male, n (%)	539 (67.5)
Age, (mean ± SD), year	73.1 ± 13.8
Race/ethnicity, n (%) *	
White Non-Hispanic	659 (84.9)
Black	67 (8.6)
Asian	26 (3.4)
Hispanic	13 (1.7)
Other	11 (1.4)
Missing	23
Body mass index (mean ± SD) *	31.7 ± 10.7
Missing	160
Diabetes, n (%)	457 (57.2)
Hypertension, n (%)	702 (87.9)

Hyperlipidemia, n (%)	511 (64.0)
Documented CAD, n (%)	465 (58.2)
Heart failure, n (%)	486 (60.8)
Ejection fraction (mean ± SD) *<br>Missing	48.4 ± 15.8<br>306
COPD, n (%)	236 (29.5)
PVD, n (%)	199 (24.9)
Asthma, n (%)	82 (10.3)
Anemia, n (%)	294 (36.8)

*For variables with missing values, proportions and p values were calculated with the non-missing data used as the denominator.

CKD: Chronic kidney disease; SD: standard deviation; CAD: Coronary artery disease; COPD: Chronic obstructive pulmonary disease; PVD: Peripheral vascular disease
